# The efficacy of different treatment approaches for pediatric OSAHS patients with mandibular retrognathia: study protocol for a multicenter randomized controlled trial

**DOI:** 10.1186/s13063-020-04398-9

**Published:** 2020-06-30

**Authors:** Yuanyuan Li, Jiali Wu, Jinghan Guo, Liming Yu, Jing Wang, Xiaoyan Li, Shuhua Xu, Min Zhu, Jinqiu Feng, Yuehua Liu

**Affiliations:** 1 Department of Pediatric Dentistry, Shanghai Stomatological Hospital, Fudan University, Shanghai, China; 2 Oral Biomedical Engineering Laboratory, Shanghai Stomatological Hospital, Fudan University, Shanghai, China; 3 Department of Otolaryngology and Head and Neck Surgery, Shanghai Children’s Hospital, Shanghai Jiao Tong University School of Medicine, Shanghai, China; 4 Department of Orthodontics, Shanghai Stomatological Hospital, Fudan University, Shanghai, China; 5 Department of Oral and Craniomaxillofacial Surgery, Shanghai Ninth People’s Hospital, College of Stomatology, Shanghai Jiao Tong University School of Medicine, Shanghai, China; 6 National Clinical Research Center for Oral Diseases, Shanghai, China; 7 Shanghai Key Laboratory of Stomatology & Shanghai Research Institute of Stomatology, Shanghai, China

**Keywords:** Pediatric obstructive sleep apnea/hypopnea syndrome, Mandibular retrognathia, Adenotonsillectomy, Orthodontic treatment, Multicenter randomized controlled trial

## Abstract

**Background:**

Pediatric obstructive sleep apnea/hypopnea syndrome (OSAHS) is a multifactorial syndrome caused by many risk factors, such as craniofacial anomalies, adenotonsillar hypertrophy, obesity, and airway inflammation. Although new treatment patterns have recently been proposed, treatment methods for children remain particularly challenging and controversial. This randomized controlled trial was designed to investigate the efficacy of adenotonsillectomy and/or orthodontic treatment for children who have mild OSAHS with mandibular retrognathia.

**Methods:**

A sample of 352 children with mild OSAHS and mandibular retrognathia, who are aged between 7 and 10 years, will be enrolled in the study. They will be randomized into four groups: the drug treatment group, the surgical treatment group, the orthodontic treatment group, or the surgery and postoperative orthodontic group. After randomization the children will receive treatments within 4 weeks. Outcome assessment will take place at the following points: (1) baseline, (2) 7 months after the treatment starting point, (3) 12 months after the treatment starting point, and (4) 24 months after the treatment starting point. The primary endpoint of the trial is the mean change in obstructive apnea/hypopnea index. Other endpoints will consist of the lowest oxygen saturation, apnea index, and hypopnea index assessed by polysomnography, subjective symptoms (assessed by the OSA-20 questionnaire), cephalometric measurements, and morphologic analysis of the upper airway.

**Discussion:**

The results of this study will provide valuable evidence for the merits and long-term efficacy of different treatment approaches and contribute to facilitating the multidisciplinary treatment of pediatric OSAHS.

**Trial registration:**

ClinicalTrials.gov: NCT03451318. Registered on 2 March 2018 (last update posted 19 April 2018).

## Administrative information

Note: the numbers in curly brackets in this protocol refer to SPIRIT checklist item numbers. The order of the items has been modified to group similar items (see http://www.equator-network.org/reporting-guidelines/spirit-2013-statement-defining-standard-protocol-items-for-clinical-trials/).

**Table:** 

Title {1}	The efficacy of different treatment approaches for pediatric OSAHS patients with mandibular retrognathia: study protocol for a multicenter randomized controlled trial
Trial registration {2a and 2b}.	Registry name: A Multicenter Study of Establishing the Multi-disciplinary Cooperative Diagnosis and Treatment Process and Evaluation System for Children with Sleep Disordered Breathing and Malocclusion Trial identifier: NCT03451318
Protocol version {3}	Version 1.0 Issue date: 17 November 2017
Funding {4}	This study was supported by the Project of Shanghai Hospital Development Center (grant number 16CR2044B) and the Project of Shanghai Municipal Health and Family Planning Commission (grant number 20184Y0018).
Author details {5a}	1, Department of Pediatric Dentistry, Shanghai Stomatological Hospital, Fudan University, Shanghai, China. 2, Oral Biomedical Engineering Laboratory, Shanghai Stomatological Hospital, Fudan University, Shanghai, China 3, Department of Otolaryngology and Head and Neck Surgery, Shanghai Children’s Hospital, Shanghai Jiao Tong University School of Medicine, Shanghai, China. 4. Department of Orthodontics, Shanghai Stomatological Hospital, Fudan University, Shanghai, China. 5. Department of Oral and Craniomaxillofacial Surgery, Shanghai Ninth People’s Hospital, College of Stomatology, Shanghai Jiao Tong University School of Medicine; National Clinical Research Center for Oral Diseases; Shanghai Key Laboratory of Stomatology& Shanghai Research Institute of Stomatology, Shanghai, China
Name and contact information for the trial sponsor {5b}	1. Shanghai Hospital Development Center. Address: No.2 Kangding Road, Shanghai, China. Email: greatshenkang@163.com 2. Shanghai Municipal Health and Family Planning Commission. Address: No.300 Shibo village road, Shanghai, China. Email: kygl@shdrc.org.
Role of sponsor {5c}	The study execution, data management, statistical analysis, and publication of the results will be performed independently from the funding sources.

## Introduction

### Background and rationale {6a}

Obstructive sleep apnea/hypopnea syndrome (OSAHS) is a sleep disorder characterized by recurrent narrowing or collapse of the upper airway (UA), resulting in sleep fragmentation and multiple episodes of apnea and/or hypopnea [1]. Pediatric and adult OSAHS share a similar pathophysiology, i.e., a recurrent reduction or cessation of airflow caused by the narrow anatomic structure and defective function of the UA. However, they are actually different disease categories due to their different pathogeneses [2]. Adenotonsillar hypertrophy is currently the major cause of pediatric OSAHS, while in adults, the major risk factor may be obesity [3, 4].

The prevalence of pediatric OSAHS is reported to be between 2 and 10% in different countries [5]. Children with OSAHS may have a variety of problems, such as cardiovascular disorders, metabolic interferences, cognitive dysfunction, and attention problems [6–9].

Conventional treatments for OSAHS include adenotonsillectomy (AT), orthodontic treatment, continuous positive airway pressure (CPAP), medication, and weight loss [10]. At present, drug therapy is mainly applied in mild OSAHS or as a complementary method for other treatments [11, 12]. For severe OSAHS or obesity, medical management has clear limitations. CPAP, which expands the UA but does not regulate the underlying mechanisms of disease, was suggested as an effective treatment [13, 14]. CPAP has been used in OSAHS treatments for many years, but its clinical application is greatly limited by poor compliance [15]. It has been reported that 46–83% of adult patients cannot adhere to treatment when 4 h of nightly use is required [16]. For children, CPAP adherence varies between studies, but the application of CPAP at night is not optimistic due to their longer sleep hours. [17, 18]. Besides, additional data show that CPAP masks can have an adverse impact on the craniofacial development of a child, which means aggravating maxillofacial deformities and leading to increased collapsibility of the UA [19].

As the main reason for pediatric OSAHS is adenotonsillar hypertrophy, the primary treatment in children has always been AT, even though many studies have suggested that the efficacy of this treatment method may not be as favorable as expected [20]. Recent reports have confirmed that the efficacy of AT varies from 27.2 to 82.9% [21–23]. Recent evidence suggested that AT could ameliorate OASHS, but that the residual apnea/hypopnea index (AHI) may persist in some cases, especially in obese children [24, 25]. Orthodontic treatment has been widely used in recent years as an alternative or combination therapy with AT.

Craniofacial deformity has an obvious influence on the collapsibility of the UA [26, 27]. It can be a primary pathogenesis of OSAHS as well as a complication caused by long-term abnormal mouth breathing [28]. Mouth breathing is one of the main clinical symptoms of OSAHS in children, and it is common to find OSAHS among mouth breathers [29]. Mouth breathing during growth may alter the muscle tone of the oropharynx, which affects the development of maxillofacial structures and presents long faces, maxillary constriction, high arched palates, and mandibular retrognathia [28, 30]. The elimination of obstructive factors is the basis for nasal respiration and normal growth of maxillofacial bone and dentition [31]. Some cohort studies have observed that AT has the potential to block the progression of craniofacial malformation [32]. However, the roles of AT in dentofacial growth were found to be limited [15, 16, 33, 34] and could be achieved only if an AT was performed before 6 years of age [34, 35]. Dentofacial deformity was more unlikely to reverse spontaneously after AT surgery for children in the mixed dentition stage [36].

Therefore, clinical workers wondered whether it was necessary for all children with OSAHS to go to an orthodontist. Rapid maxillary expansion (RME) and mandibular advancement devices (MADs) are the most commonly used appliances for children with OSAHS [37–41]. RME benefits children with OSAHS through the following mechanisms: (1) it enlarges the dimension of the nasal cavity and increases nasal respiration; (2) it increases the maxillary width so that a better tongue position can be induced; (3) the normal width of the dental arch stimulates the development of the lower jaw. Camacho et al. stated in a systematic review that RME has stable long-term efficacy for pediatric OSAHS patients with transverse maxillary deficiency or narrow hard palates [39]. Villa et al. assessed the outcome of AT and RME in a non-randomized controlled trial and found that both of these treatment methods were effective, but further studies were needed to evaluate their long-term efficacy [42].

MADs, in the form of oral appliances, can promote the anterior displacement of the mandible and hyoid bone, leading to anterior traction of the tongue and thus an enlarged UA dimension. Pavoni et al. found that after MAD treatment, significant improvements in sagittal airway dimensions, hyoid position, and tongue position were induced, and obvious relief in subjective symptoms was observed in children with sleep-disordered breathing [43]. Many studies have reported that the clinical use of functional therapy for mandibular advancement such as twin-block and Frankel appliances significantly reduced AHI in patients with OSAHS [10, 40, 41]. In recent years, the combination of RME and MADs was illustrated by some clinicians.

For OSAHS children with adenotonsillar hypertrophy and dentoskeletal Class II malocclusions, who represent a large proportion of pediatric OSAHS patients, both orthodontic treatment and AT may be effective, but little evidence-based medical research has been found to our knowledge. There have been longstanding debates among clinicians about the indications for AT, especially for children with mild OSAHS. More convincing evidence is needed to prove that AT/orthodontic treatment has a therapeutic effect for children with OSAHS and that it improves the craniomaxillofacial deformity caused by mouth breathing.

### Objectives {7}

This clinical trial was designed to investigate the efficacy of AT and/or orthodontic treatment for OSAHS children with mild mandibular retrognathia. The efficacy will be evaluated by the improvement in subjective symptoms, polysomnography (PSG) data, UA structure, and maxillofacial development.

### Trial design {8}

**Fig. 1:**
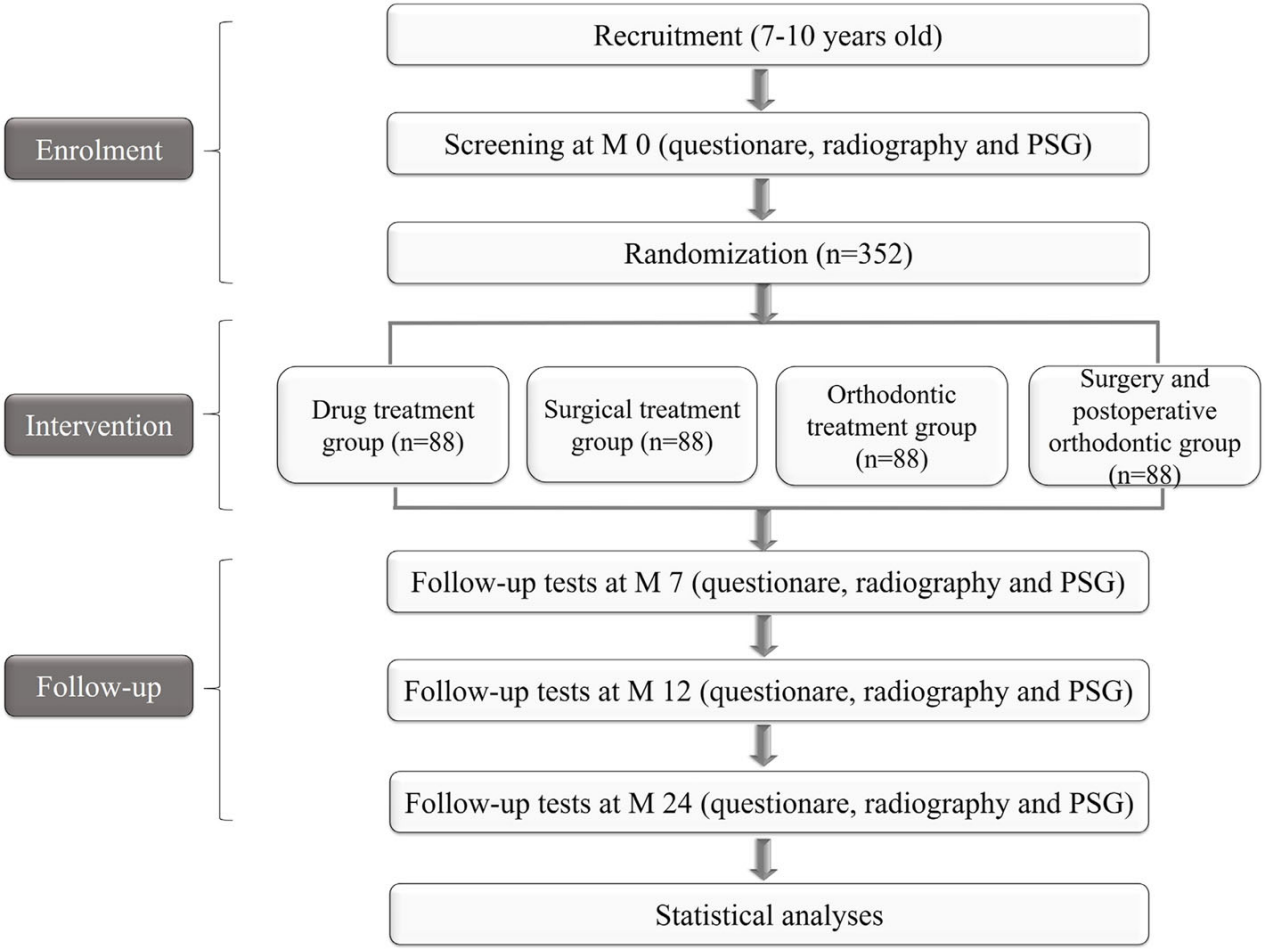
Flow chart of study design

This study will be a multicenter and randomized controlled pilot trial. Clinical research coordinators will explain the purpose and the procedure of this study to potential subjects. The participants will undergo a series of medical tests, which will include physical examinations, maxillofacial radiography, and polysomnography, to verify the diagnosis of OSAHS and malocclusion. Subjects will be randomly divided into one of four groups—the drug treatment group, the surgical treatment group, the orthodontic treatment group, or the surgery and postoperative orthodontic group—at a ratio of 1:1:1:1. Regardless of group assignment, tests will be conducted on each group before the treatment (M 0), 7 months after the treatment (M 7), 12 months after the treatment (M 12), and 24 months after the treatment (M 24). Figure 1 shows the flow chart of the research, and Table 1 shows the clinical trial schedule.

**Table 1:** Schedule of enrollment, interventions, and outcome assessment

Action/timepoint	Enrollment	Baseline survey (M 0)	Randomization	Treatment	Follow-up (M 7)	Follow-up (M 12)	Follow-up (M 24)
Informed consent	⚪						
Demographic characteristics	⚪						
Medical history	⚪						
Physical examination	⚪						
Questionnaire (OSA-20)	⚪						
Lateral cephalometrics		⚪			⚪	⚪	⚪
Polysomnogram		⚪			⚪	⚪	⚪
Confirm suitability for study		⚪					
Allocation			⚪				
Drug treatment				⚪			
Surgical treatment				⚪			
Orthodontic treatment				⚪			
Surgery and postoperative orthodontic treatment				⚪			
Assessment							
Symptom changes					⚪	⚪	⚪
Questionnaire (OSA-20)		⚪			⚪	⚪	⚪
Cephalometric measurements		⚪			⚪	⚪	⚪
Morphologic analysis of UA		⚪			⚪	⚪	⚪
Polysomnogram		⚪			⚪	⚪	⚪
Safety assessment					⚪	⚪	⚪

## Methods: participants, interventions, and outcomes

### Study setting {9}

Participants will be recruited at Shanghai Stomatological Hospital, Shanghai Ninth People’s Hospital affiliated to Shanghai Jiao Tong University, and Shanghai Children’s Hospital, all of which are located in Shanghai, China, from May 2018 to December 2021 (anticipated). These hospitals in Shanghai comprise relevant diagnostic and treatment departments required for this study, including ear, nose, and throat and stomatological departments. Notification of subject recruitment will be published in these three hospitals and on their official websites.

### Eligibility criteria {10}

All participants will be assessed by a treatment group consisting of more than three experienced pediatric physicians, otolaryngologists, and orthodontists.

#### Inclusion criteria

The inclusion criteria for the study are as follows:

1. Patients aged 7–10 years
2. Patients diagnosed with mild OSAHS (an apnea index of 1–5 events per hour according to PSG)
3. Patients with hypertrophy of the tonsils or adenoids
4. Patients with mouth breathing
5. Patients with constricted dental arch or mandibular retraction (A point, nasion, B point [ANB] ≥ 4.5)
6. Patients whose guardians agree to enter this trial and sign the relevant informed consent form.

#### Exclusion criteria

Patients will be excluded from the trial if they:

1. Are diagnosed with central sleep apnea/hypopnea syndrome
2. Have concurrent systemic diseases
3. Have rhinostenosis
4. Have a z score equal to or greater than 3 based on the body mass index (BMI).

### Who will take informed consent? {26a}

A qualified clinical research assistant will obtain written informed consent from the guardians of participants after full explanation of this study.

### Additional consent provisions for collection and use of participant data and biological specimens {26b}

On the consent form, guardians will be asked if they agree to the use of their children’s data should they choose to withdraw from the trial. They will also be asked for permission for the research team to share relevant data with people from the universities taking part in the research or ethics committees or regulatory authorities, where relevant. This trial does not involve collecting biological specimens for storage.

## Interventions

### Explanation for the choice of comparators {6b}

Due to poor compliance, the application of CPAP for pediatric OSAHS is decreasing in the clinic. The most commonly used treatments are medication, AT, and orthodontic treatment. For children with mild OSAHS and mandibular retrognathia, both orthodontic treatment and AT may be effective in terms of PSG data and maxillofacial development, but there is no evidence-based medical research to compare the efficacy of AT and/or orthodontic treatment. Furthermore, we wondered what the indications are for the use of these two therapies, individually or in combination. Thus, in this study, the comparators included a drug treatment group, a surgical treatment group, an orthodontic treatment group, and a surgery and postoperative orthodontic group.

### Intervention description {11a}

All subjects will receive different treatments, i.e., drug treatment, surgical treatment, orthodontic treatment, and surgery or postoperative orthodontic treatment, within 4 weeks after randomization. The practitioners will be doctors who have a minimum of 5 years of work experience in their hospital and have undergone training in the trial protocol prior to the study. The training mainly includes information on the recruitment and randomization of subjects, the standardized treatment procedures, the completion of the case report form (CRF), and the adverse event reporting system.

Drug treatment group: Patients in this group will receive mometasone furoate aqueous nasal spray once a day (0.1 mg) in the morning for 2 months. The research assistants will interview the participants’ parents by telephone to remind the patients of the treatment stage.

Surgical treatment group: Patients in this group will receive AT under general anesthesia. Routine follow-up will be conducted 2 weeks after surgery to evaluate the surgical effects and the prognosis.

**Fig. 2:**
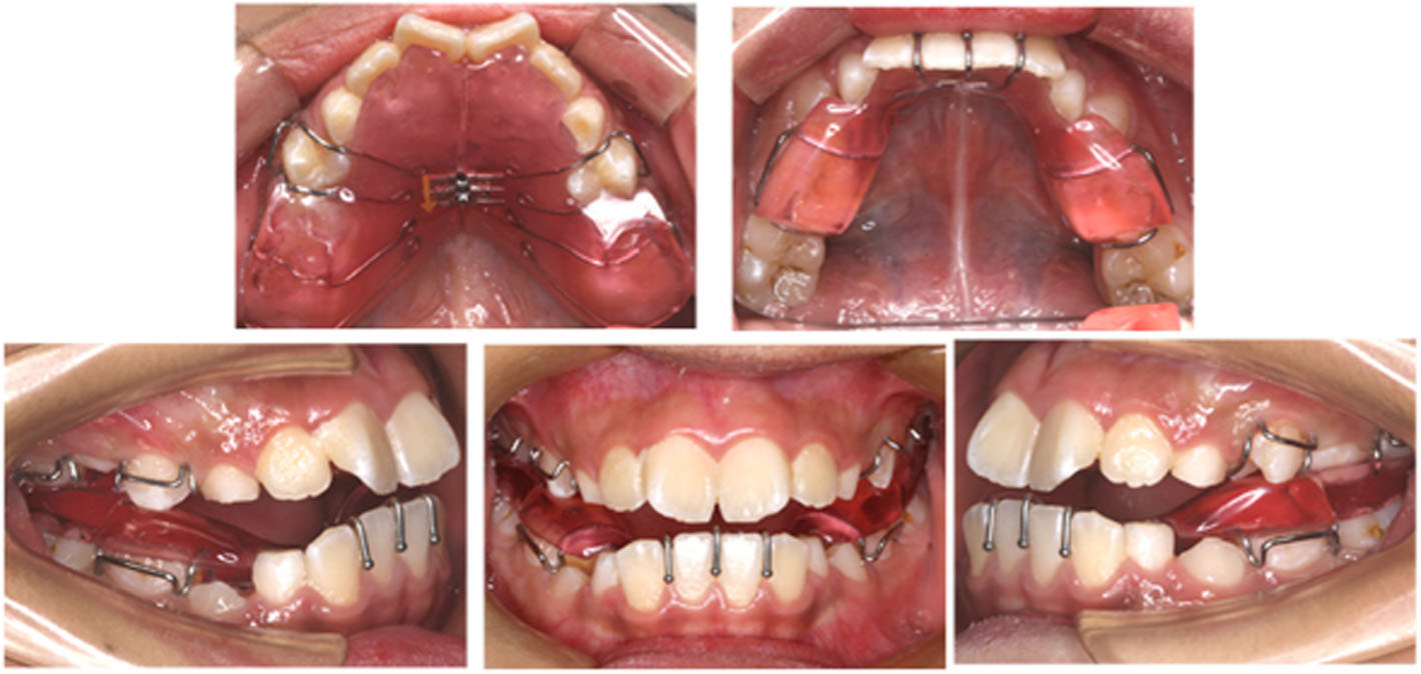
The removable twin-block appliance combined with RME used in the orthodontic treatment

Orthodontic treatment group: Patients in this group will receive orthodontic treatments. They will receive orthodontic treatment according to a consistent comprehensive protocol, which mainly involves the use of a removable twin-block appliance combined with RME (see Fig. 2).

Surgery and postoperative orthodontic group: Patients in this group will receive AT under general anesthesia and subsequent orthodontic treatment after surgery. The orthodontic treatment appliance will also be a removable twin-block appliance combined with RME.

### Criteria for discontinuing or modifying allocated interventions {11b}

Participants may quit the study at their guardians’ discretion or defer to the supervisory team. Circumstances that will suspend participants from the study include adverse events and detection of evidence that may influence the outcomes of the research. Subjects who leave the study will be followed up until they are in stable status.

### Strategies to improve adherence to interventions {11c}

Additional services will be provided for participants through WeChat or phone to arrange the treatment time to enhance their adherence. The monitor or principal researcher will visit every study site monthly to ascertain that all aspects of the program are being followed and that the trial is being carried out in accordance with the provisions of the drug clinical trial quality management good clinical practice (GCP). During the monitoring visit, the monitor will check the CRF for subjects in the trial to confirm that all items have been completed and that the data are being obtained according to the protocol. The monitor will also check that the CRF data are consistent with the clinical records or the original data.

### Relevant concomitant care permitted or prohibited during the trial {11d}

Other treatments that may affect the outcomes of the study, such as traditional Chinese medicine, acupuncture, and massage, are prohibited during the trial.

### Provisions for post-trial care {30}

Participants may quit the study at their guardians’ discretion or defer to the supervisory team if they are not responding well to treatment. They will be followed up and receive appropriate therapy free of charge after they leave the study.

### Outcomes {12}

Endpoint measurements will be performed before the treatment (baseline survey; month 0) and at 7 months (M 7), 12 months (M 12), and 24 months (M 24) after the starting point.

#### Primary endpoint

The primary endpoint of the trial is the mean change in AHI from baseline (M 0) to the primary endpoint (M 7), because PSG is still the gold criterion for diagnosing OSAHS. All subjects will undergo attended overnight PSG in a hospitalized ward. They will go to bed at their usual sleeping time and sleep for at least 6 h to ensure PSG monitoring time. A reduction in airflow of more than 90% compared to that preceding sleep breathing is considered to be an obstructive apnea. A hypopnea is defined as a reduction in airflow of more than 30%, accompanied with an oxygen desaturation of ≥ 3% and/or an arousal. A duration of the obstructive apnea or hypopnea equal to or greater than two respiratory cycles is defined as an obstructive event. The definition of AHI is the number of obstructive events per hour. The analysis will include mixed events but not central events.

#### Secondary endpoints

The secondary endpoints will consist of the lowest oxyhemoglobin saturation (LSaO_2_), apnea index, and hypopnea index assessed by PSG, subjective symptoms (assessed by the OSA-20 questionnaire), cephalometric measurements, and morphologic analysis of the UA.

#### Subjective symptoms

Subjective symptoms will be assessed according to the OSA-20 questionnaire. OSA-20 includes 20 questions divided into five domains: sleep interference, physical suffering, emotional disorder, diurnal problems, and guardian concern. These questions are graded on a scale of 1 to 7 applying an ordinal Likert scoring system. Guardians will complete the questionnaire without help to ensure its reliability and validity. A total score ranging from 20 to 140 points will be associated with quality of life.

#### LSaO_2_

The lowest oxyhemoglobin saturation will be assessed according to PSG.

#### Cephalometric measurements

**Fig. 3:**
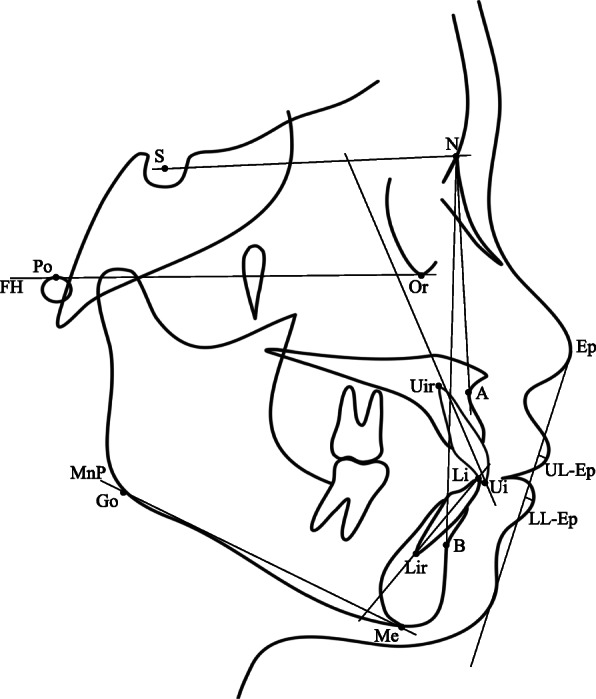
Cephalometric landmarks and indicators. *S* sella, *N* nasion, *Po* porion, *Or* orbitale, A subspinale, B supramental, *Ui* upper incisor, *Uir* upper incisor root, *Li* lower incisor, *Lir* lower incisor root, *Me* menton, *Go* gonion, *UL* upper lip anterior, *LL* lower lip anterior, *FH* the Frankfort plane, *MnP* the mandibular plane, *Ep* the aesthetic plane, *UL-Ep* distance between UL and the aesthetic plane, *LL-Ep* distance between LL and the aesthetic plane

Lateral cephalometric radiographs will be taken for all the subjects at the intercuspal occlusion and then traced and digitized using the software Dolphin Imaging (Dolphin Imaging & Management Solutions Corporation, Chatsworth, CA, USA). The cephalometric measurements are presented in Fig. 3. The measurement items are as follows: SNA, angular indicator for assessment of maxillary protrusion; SNB, angular indicator for assessment of mandibular protrusion; ANB, angular indicator for assessment of the sagittal relationship between the jaws; MP-FH, angle between the mandibular plane and Frankfort plane; MP-SN, angle between the mandibular plane and SN plane; UI-SN, angular indicator for assessment of upper anterior teeth protrusion; LI-MP, angular indicator for assessment of lower anterior teeth protrusion; UL-Ep, distance between upper lip and the aesthetic plane; LL-Ep, distance between lower lip and the aesthetic plane.

#### Morphologic analysis of UA

**Fig. 4:**
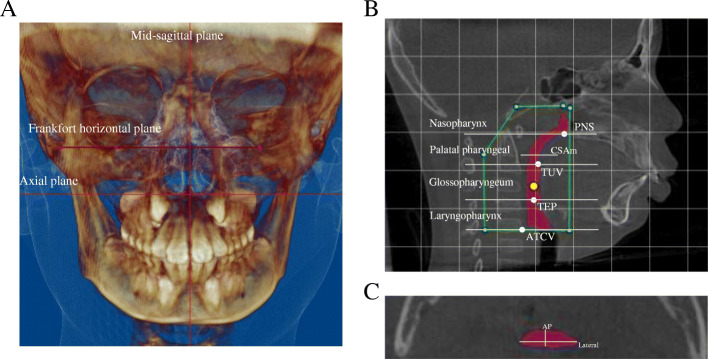
Morphologic analysis of upper airway. **a** Localization planes of CBCT images. **b** Segmented upper airway. **c** Minimum cross-sectional area (*CSAm*) on the axial slice of the CBCT image. *PNS* posterior nasal spine, *TUV* tip of the uvula, *TEP* tip of the epiglottis, *ATCV* anteroinferior aspect of the vertebral body of the third cervical vertebra, *AP* anteroposterior

Cone beam computed tomography (CBCT) of subjects will be performed at the Radiology Department of Shanghai Stomatological Hospital according to the standard protocol. During the scanning, subjects will be positioned in orthostatism, with the Frankfort plane parallel to the floor. The images will be digitized in Digital Imaging and Communications in Medicine (DICOM) files and then imported into Dolphin software for anatomic landmark localization and UA measurements. Two orthodontists will be trained as analysts and will perform the localization and UA measurements as presented in Fig. 4. The analyst will be blinded to patients’ information during the measurements.

### Participant timeline {13}

The clinical trial schedule is shown in Table 1.

### Sample size {14}

The sample size was calculated using SAS 9.4 (SAS Institute Inc., Cary, NC, USA) in this study. Our expectation hypothesis is that the intervention may decrease subjects’ AHI. In a study reported in 2016, the AHI score decreased by 0.8 in children with mild OSAHS who received AT, while it increased by 2.1 in the watchful waiting group (*P* < 0.05) [44]. Another non-randomized study reported that the average AHI of children decreased by 2.0, 15, and 3.7 after medical therapy, AT, and orthodontic treatment respectively [45]. If 80 eligible subjects in each group are expected, we will be able to detect an effect value of 0.4 (the average decreasing of AHI among groups was 2.9) at a power of 80% and a 5% significance level. In consideration of a potential dropout rate of approximately 10%, a total sample size of 352 subjects will be required in this study.

### Recruitment {15}

Participants will be recruited at Shanghai Stomatological Hospital, Shanghai Ninth People’s Hospital affiliated to Shanghai Jiao Tong University, and Shanghai Children’s Hospital, all of which are located in Shanghai, China, from May 2018 to December 2021 (anticipated). These hospitals comprise all diagnostic and treatment departments, including ear, nose, and throat and stomatological departments. Notification of subject recruitment will be published in these three hospitals and on their official websites.

## Assignment of interventions: allocation

### Sequence generation {16a}

A Central Randomization System was designed with the help of Shanghai KNOWLANDS MedPharm Consulting Co., Ltd., and then installed in researchers’ phones in every study site.

### Concealment mechanism {16b}

The Central Randomization System can be used for the management of participant information and randomization. Prior to signing an informed consent form, this study will be fully explained to subjects and their guardians by researchers at every study site. The basic information of patients will be registered into the Central Randomization System by research assistants. After the baseline survey, the eligible participants will be divided into four groups at random with a ratio of 1:1:1:1.

### Implementation {16c}

Randomization will be implemented by a data analyst from Shanghai KNOWLANDS MedPharm Consulting Co., Ltd., using the minimization method with 80% allocation probability according to the following stratification factors: (1) male/female patient; (2) obese or not based on BMI. Then an email will be submitted automatically to the project coordinator with the ID numbers and groups of participants to be randomized. The project coordinator will then contact participants to inform them about the initiation of treatment.

## Assignment of interventions: blinding

### Who will be blinded? {17a}

When analyzing the outcomes, the analyst will be blinded to the patients’ information during the measurements.

### Procedure for unblinding if needed {17b}

This item is not applicable. The analysts are not allowed to be unblinded under any circumstances.

## Data collection and management

### Plans for assessment and collection of outcomes {18a}

Case report forms (CRFs) specially designed by coordinator investigators and Shanghai KNOWLANDS MedPharm Consulting Co., Ltd. are used for documentation. The training related to recruitment, screening, randomization, evaluation, and instructions for CRF will be organized before the study for the researchers in order to enable conduction of a high-quality clinical trial. A CRF must be filled out for every subject by investigators. All data and information in the CRF must be clear. Changes in the CRF must be marked with a line across the incorrect data. Both incorrect data and the corrections must be kept legible, and the signature of the researcher and time of correction must be marked next to corrected data. A qualified clinical research assistant who is blinded to the patients’ group will supervise the study process at a fixed period, including the completion of informed consent, the screening process, the intervention treatment, the recording of adverse events, and the filling out of the CRF. The original CRFs will be used by the data management center and then returned to the sponsor center, Shanghai Stomatological Hospital.

### Plans to promote participant retention and complete follow-up {18b}

Clinical research assistants from every study site will contact participants monthly to promote their retention and complete their follow-up. Additional services will be provided for participants through WeChat or phone to arrange treatment times to enhance adherence.

### Data management {19}

The original CRFs will be used by the data management center and then returned to the sponsor center, Shanghai Stomatological Hospital. All the questionnaires, PSG data, and image measurement data will be collected, stored in separate folders, and regularly checked. The outcomes will be encoded and entered in a timely manner using EpiData 3.1 double-entry test results.

### Confidentiality {27}

Subjects will be informed that their data will be stored and analyzed on a computer, but that only the research team members can obtain the information associated with each subject. In addition, subjects will be informed of the possibility that their clinical records will be reviewed by representatives of the sponsor and/or regulatory authority. All unpublished information, including patent applications, production processes, and basic data, will be deemed confidential. Any public report of the results of this study will not disclose the personal identity of the participants. The trial data will be available for public inquiry and sharing, which will be limited to web-based electronic databases to ensure that no personal privacy information is disclosed.

### Plans for collection, laboratory evaluation, and storage of biological specimens for genetic or molecular analysis in this trial/future use {33}

This item is not applicable. No samples will be collected.

## Statistical methods

### Statistical methods for primary and secondary outcomes {20a}

Statistical analysis will be conducted using IBM SPSS Statistics (version 22.0). Descriptive analysis will be performed for all of our variates. The analysis set will consist of the intention-to-treat (ITT) set, per protocol (PP) set, and safety set. All subjects will be included in the ITT set after randomization regardless of whether they received the treatment or not. Subjects who complete treatment sessions (M 0 to M 7) without any major violation to the trial procedure will be included in the PP set. Participants who receive treatment will be included in the safety set for safety analysis. The significance level will be set so that *P* < 0.05 will be considered significant.

The primary endpoint of this study is the change in AHI from M 0 to M 7. Intergroup comparisons will be performed using an analysis of covariance (ANCOVA) to adjust for gender, obesity status, and baseline AHI. The outcomes will be mainly analyzed using the PP set. Linear regression will be used to evaluate the correlation between observed differences of AHI and the potential prognostic factors, such as gender, BMI, neck size, and research site.

### Interim analyses {21b}

No interim analysis is planned in the study.

### Methods for additional analyses (e.g., subgroup analyses) {20b}

The analysis of LSaO_2_, the volume of the UA, and the total score of OSA-20 in secondary endpoints will use similar statistical methods as those used for the AHI. Descriptive statistical analysis will be used for the measurements of lateral cephalometric film and UA.

### Methods in analysis to handle protocol non-adherence and any statistical methods to handle missing data {20c}

Missing data will be dealt with using the last-observation-carried-forward (LOCF) method.

### Plans to give access to the full protocol, participant-level data, and statistical code {31c}

The datasets analyzed during the current study will be available from the corresponding author on reasonable request.

## Oversight and monitoring

### Composition of the coordinating center and trial steering committee {5d}

The monitor team will visit every study site biannually to ascertain that all aspects of the program are being followed and that the trial is being carried out in accordance with the provisions of the drug clinical trial quality management practices (GCP). There are three research centers in the study. Each research center includes at least three main investigators and one coordinator. Clinical research coordinators will be responsible for registering information, obtaining informed consent, and keeping in contact with participants to inform them about treatment procedures. The investigators are experienced doctors who will perform examinations and interventions for the patients. A supervisory team of three experienced doctors will be established to assess and manage the adverse events. The data processing team includes two orthodontists who will perform the localization and UA measurements and three statisticians.

### Composition of the data monitoring committee, its role and reporting structure {21a}

A data monitoring committee is not applicable. Intervention measures in the study are well established and commonly used treatment methods, with almost no safety problems.

### Adverse event reporting and harms {22}

Adverse events will be recorded and reported by researchers within 24 h. A supervisory team of three experienced doctors from every research site will be established to assess and manage the adverse events. Serious adverse events will be reported to the Ethics Committee. For subjects in the drug and surgical treatment groups, the observation time for reporting adverse events will be from M 0 to M 7; for subjects who receive orthodontic treatment, the observation time will be from M 0 to the end of orthodontic treatment. Participants may quit the study at their guardians’ discretion or defer to the supervisory team. Circumstances that will suspend participants from study include adverse events and detection of evidence that may influence the outcomes of research. Furthermore, subjects who leave the study will be followed up until they are in stable status.

### Frequency and plans for auditing trial conduct {23}

The monitor team, consisting of people from the sponsor Shanghai Hospital Development Center, will visit every study site biannually to ascertain that all aspects of the program are being followed and that the trial is being carried out in accordance with the provisions of the drug clinical trial quality management practices (GCP). During the monitoring visit, the monitor will check the CRFs for subjects in the trial to confirm that all items have been completed and that the data are being obtained according to the protocol. The monitor will also check that the CRF data are consistent with the clinical record or the original data.

### Plans for communicating important protocol amendments to relevant parties (e.g., trial participants, ethical committees) {25}

During the study, there may be program modifications that may affect the study or patient safety. Any modifications shall be agreed upon by the sponsor/researcher and the Ethics Committee prior to their implementation. Revisions shall be recorded in a new written version, which must be signed by the same parties who signed the final draft of the pilot protocol.

### Dissemination plans {31a}

The final clinical report will be the basis for the study to be published in a medical journal or reported at conferences. A formal report or publication of the data from the study will be jointly published by a person appointed by the principal investigators.

## Discussion

Cranial and maxillofacial growth and development in children are influenced by both genetic and environmental factors [46]. The change of respiratory mode in children may alter the oropharyngeal muscle tone, which affects the development of maxillofacial sections. Mouth breathing is one of the main clinical symptoms of OSAHS in children, and it is common to find facial features such as long faces, maxillary constriction, high arched palates, and mandibular retrognathia among mouth breathers [28, 30]. The contraction of the upper airway (UA) is closely related to this kind of maxillofacial deformity, while maxillofacial deformities can also be the primary or promoting factor for OSAHS. The longer mouth breathing continues and the more serious the skeletal alterations that occur in OSAHS children, the more difficult it will be to cure the OSAHS. Therefore, early diagnosis of the morphologic analysis of the UA and maxillofacial structure is crucial for pediatric OSAHS. In this study protocol, CBCT of subjects will be performed for both morphologic analysis of the UA and cephalometric measurements to assist in diagnosis and efficacy evaluation. Traditional and commonly used efficacy evaluation methods in pediatric OSAHS such as PSG and the OSA-20 questionnaire are also included in this study.

Although new treatment patterns have recently been proposed, treatment methods for children have continued to be particularly challenging [26]. Furthermore, there is still great controversy concerning the treatment of OSAHS in clinic due to the lack of randomized controlled studies comparing treatment approaches. In this study, a multicenter randomized controlled trial was designed to investigate the advantages and disadvantages of AT and/or orthodontic treatment for children with mild OSAHS and mandibular retrognathia.

This study protocol still faces several limitations and challenges. The first is the challenge of sample selection of children with mild OSAHS and mandibular retrognathia. Due to the limited acceptance of randomization, it may take a long time to collect the sample size required for this study. The second is the challenge of subjects’ compliance. Additional services will be provided for participants through WeChat or phone to arrange treatment times and enhance adherence. The third limitation is the consistency of treatment. Before the study begins, all of the surgical and orthodontic treatment teams will receive specific training on the normalized research procedure to ensure consistency.

The results of this study will provide valuable evidence for the merits and long-term efficacy of different treatment approaches and contribute to facilitating the multidisciplinary treatment of pediatric OSAHS.

## Trial status

The study has been ongoing from May 2018. Recruitment is expected to be completed by the end of December 2021. The protocol version is 1.0 (issue date 17 November 2017).

## Abbreviations

AHI: Apnea/hypopnea index
AI: Apnea index
AT: Adenotonsillectomy
BMI: Body mass index
CBCT: Cone beam computed tomography
CPAP: Continuous positive airway pressure
CRA: Clinical research assistant
CRF: Case report form
HI: Hypopnea index
ITT: Intention-to-treat (set)
LSaO_2_: Lowest oxyhemoglobin saturation
MAD: Mandibular advancement device
OSAHS: Obstructive sleep apnea/hypopnea syndrome
PP: Per protocol (set)
RME: Rapid maxillary expansion
